# Do Dentists Require Leadership Qualities?

**Published:** 2012-03-31

**Authors:** Mamata Hebbal, Anil Ankola, Vikneshan Murugaboopathy, Sagar Patel, Niharika Patel, Nayeema Parkar

**Affiliations:** KLE VK Institute of Dental Sciences, Belgaum, India

Leadership generally entails sustaining, improving, or changing strategic directions within small or large, simple or complex, organizations.^1^ Today’s era raises issues for leadership that focus on reducing social and health benefits, and downsizing workforces and related services. Leadership requires making choices with finite resources based upon plausible alternatives, and it depends on motivating people and bringing them along, on mobilizing and coordinating human and financial resources to achieve a common goal.^1^

Today, opportunities abound for activities related to healthcare leadership worldwide.^2^ Currently, leadership is most needed to envision the future, to reallocate resources, to monitor progress using information technology,^2^ and to produce both evidence-based as well as outcome-based healthcare for all.

Communities that have excessive burdens of disease based upon socioeconomic determinants, require a culturally competent healthcare workforce. It is also believed that creating links within and between health professional schools, departments, and programs will be the key to prepare the healthcare workforce to meet the changing health needs of society.^3^ An effective leader is required for the proper functioning of this healthcare force. In the field of dentistry, effective leaders are needed to move the dental profession forward, building on past accomplishments, meeting new challenges, and leading innovation and change. Leadership is also an important attribute for practitioners, as the dentists becomes the leaders of their team.^4^

In recent years, there has been increasing interest in developing dentistry’s future leaders. This is clearly confirmed by the growing number of leadership development programs created for dental professionals. Leadership development at an early stage in the career has received less attention, particularly for dental students in their undergraduate years. The period in dental school presents one of the best opportunities for early identification and purposeful development of leadership talent, interest, and abilities. There is a lack of research regarding students’ perceptions of the importance of leadership abilities and/or their interest in developing leadership skills in dental school and this will have important implications for program design.

A survey was conducted in an Indian dental institute to explore dental student’s perceptions regarding leadership development. The students strongly agreed that it is important for dentists to have leadership skills and that they have the potential to become leaders. Many students strongly disagreed that leaders are born and not made. Students agreed that leadership is a skill that can be learned and many also agreed that dentists should assume a leadership role in general health related events.

Dentists should not only look into oral health matters, but should also take an active role in other issues where the health of the population is at risk. Leaders play an active role in the community and they have to be an example to others.^5^ In this process, leaders may have to make compromises in their personal life and give priority to their leadership role in the community. They may face many obstacles as they have to make important decisions that may go against the opinions of others.^6^

In India, it is not uncommon for students to concentrate solely on academic activities. The trend is changing, however, and the younger generation is more oriented towards taking responsibilities and participating in innovative programs. The majority of students agreed that they were interested in assuming a leadership role in dental practice, academics, and dental associations in the future.

The students surveyed had a positive outlook towards leadership and the finding that leadership is considered a skill that can be learned is encouraging for dental educators to include leadership activities in the curriculum. Students expressed interest in participating in leadership programs and more efforts should be made to increase participation in these programs.

Given the current realities in the healthcare industry, the complexity, and the requirement of synergy between various healthcare fields, dentistry faces the challenge of assuming a leadership role in meeting the demands and expectations of these changing systems. It is recommended that leadership qualities be instilled early on by incorporating leadership programs in the curriculum, which will provide the dental community with leaders who can take our profession forward.
